# Apical Third Cleaning Efficiency of Hand, Rotary, and Reciprocating Root Canal Instrumentation: A Quantitative In Vitro Scanning Electron Microscopic Study

**DOI:** 10.7759/cureus.101717

**Published:** 2026-01-16

**Authors:** B. Sai Krishna, Sita Mahalakshmi Koppu, Dilip Katakam, Sahithi Nammaniwar, Ambika Belam, Akshita Balivada, Divakar K. P.

**Affiliations:** 1 Conservative Dentistry and Endodontics, Employees State Insurance Corporation (ESIC) Dental College and Hospital, Gulbarga, Gulbarga, IND; 2 Periodontology and Implantology, Atal Hospital, Bina Area, Northern Coalfields Limited (NCL), Bina, IND; 3 Conservative Dentistry and Endodontics, Tirumala Institute of Dental Sciences and Research Centre, Nizamabad, IND

**Keywords:** apical third cleanliness, debris removal, endodontic instrumentation, hand k files, nickel-titanium files, reciprocating instrumentation, root canal preparation, rotary instrumentation, scanning electron microscopy, smear layer

## Abstract

Objective: This in vitro study quantitatively evaluated the cleaning efficiency of hand, rotary, and reciprocating instrumentation systems in the apical third of root canals using scanning electron microscopy (SEM).

Materials and methods: Sixty extracted single-rooted human premolars with single canals were randomly assigned to three groups (n = 20 each): Group I: hand instrumentation using stainless steel K‑files; Group II: rotary preparation with ProTaper Next (Dentsply Maillefer, USA); and Group III: reciprocating shaping using WaveOne Gold (Dentsply Sirona, USA). Instrumentation protocols were standardized in working length, irrigation sequence (3% sodium hypochlorite (NaOCl) and 17% ethylenediaminetetraacetic acid (EDTA)), and drying. Following preparation, roots were grooved, split longitudinally, gold sputter-coated, and examined under SEM (×1500) for quantitative assessment of the apical third using ImageJ software (National Institutes of Health, Bethesda, MD, USA). Smear layer, pulpal debris, and inorganic debris were scored on a four-point scale. Data were analyzed using the Kruskal-Wallis and Mann-Whitney U tests (α = 0.05).

Results: Hand instrumentation showed significantly lower mean scores for smear layer (3.00 ± 0.92), pulpal debris (2.40 ± 0.50), and inorganic debris (2.60 ± 0.50) compared to rotary and reciprocating systems (p < 0.001). No statistical difference was observed between the rotary and reciprocating groups across evaluated parameters (p > 0.05).

Conclusion: Within the limitations of this study, hand instrumentation with stainless steel K‑files achieved superior apical cleanliness compared to ProTaper Next and WaveOne Gold systems. The effective scraping and debris dislodgment capability of hand files contributed to enhanced smear layer and debris removal in the apical third, underscoring their clinical relevance in achieving improved disinfection in complex canal anatomies.

## Introduction

The fundamental principle of endodontics emphasizes complete removal of pulpal remnants, debris, and microorganisms from the root canal system prior to obturation [1]. The most crucial steps of root canal treatment are cleaning and shaping, as cleaning eliminates bacteria, which are the primary etiology of pulpal and periapical pathology, and shaping facilitates access for irrigants. The instrumentation creates a smear layer that includes dentinal chips, odontoblastic remnants, and necrotic debris, which is pressed into dentinal tubules up to 40 µm deep, hindering the passage of irrigants and the formation of a canal seal [2,3].

Hand, rotary, and reciprocating systems are three major approaches to root canal instrumentation, each developed to enhance cleaning, shaping, and safety of endodontic treatment. Hand instruments, usually stainless steel K‑files and H‑files, are operated manually with watch winding and filing motions, offering excellent tactile feedback that helps the clinician sense canal curvature, calcification, and apical anatomy. They allow careful negotiation of narrow and curved canals but are more time-consuming and operator-fatiguing than engine‑driven systems.

Rotary nickel-titanium (NiTi) systems use motor‑driven continuous rotation to standardize and streamline canal preparation, taking advantage of NiTi superelasticity and other alloys like M-Wire or CM-Wire to shape curved canals with more uniform taper and reduced canal transportation [4]. These systems shorten instrumentation time and improve reproducibility but leave smear and debris in the apical third due to ramification challenges [5].

Reciprocating NiTi systems modify the engine‑driven concept by applying an alternating back‑and‑forth motion, often using a single or very limited number of instruments to complete canal preparation. This asymmetric reciprocation lowers cyclic fatigue on the file, decreases the likelihood of instrument fracture, and allows relatively rapid preparation while maintaining the flexibility benefits of NiTi in curved canals. However, variations in reciprocation angles, file design, and cutting efficiency among different systems can influence debris extrusion and shaping quality, so clinicians must understand the specific kinematics of the chosen reciprocating system [6].

This in-vitro study aimed to compare the cleanliness of the apical third of root canals prepared with hand, rotary, and reciprocating instruments, using scanning electron microscopy for surface evaluation. The null hypothesis stated no significant differences among techniques in smear layer scores, pulpal debris scores, and inorganic debris scores. The primary endpoints were ordinal scores for these metrics at the apical third. Scores for the three groups were planned to be compared with a non-parametric test (Kruskal-Wallis), followed by post-hoc Mann-Whitney U tests for pairwise comparisons at a 5% significance level. This allows it to address the evidence gaps with standardized methods by targeting this risky area, which enables evidence-based instrumentation decisions to achieve high-quality endodontic prognosis.

Scanning electron microscopy (SEM) provides high-resolution dentinal surface appraisal after the instrumentation, invariably disclosing residual debris across methods [7]. Literature documents hand files surpassing certain rotary systems in apical smear clearance, with reciprocation occasionally outpacing rotation; however, three-arm trials isolating smear, pulpal, and inorganic debris in the apical third are sparse, yielding variable results due to irrigant variances, canal anatomy, and protocols [8]. These discrepancies restrict reliable apical disinfection directives amid NiTi metallurgical progress [9].

To the best of our knowledge, this study is the first to quantitatively evaluate, using SEM, hand, rotary, and reciprocating files in the apical third, scoring smear layer, pulpal remnants, and inorganic debris to harmonize the contradictory data. This allows it to address the evidence gaps with standardized methods by targeting this vulnerable area, which enables evidence-based instrumentation decisions to achieve high-quality endodontic prognosis [10].

## Materials and methods

This in‑vitro study was conducted in the Department of Conservative Dentistry and Endodontics, Kamineni Institute of Dental Sciences, Hyderabad, India. Institutional Ethics Committee approval (KIDS/IEC/UG/2022/20) was obtained prior to sample collection and experimentation. The sample size of 60 teeth (20 per group) was determined using G*Power software (version 3.1.9.6; Heinrich-Heine-Universität Düsseldorf, Germany) based on the prior SEM study by Matos et al. (2020) [11] evaluating smear layer removal, assuming a standard deviation of 0.5-1.0, a minimum detectable difference of 0.7-1.0 scores between groups, α = 0.05, and power = 0.80-0.90 to detect significant differences in debris scores across instrumentation methods. Sixty single-rooted, single-canal human permanent premolar teeth extracted for non-endodontic reasons were selected after radiographic verification (buccolingual and mesiodistal views) to confirm fully formed apices and absence of calcifications, cracks, caries, internal/external resorption, or prior treatment. The teeth were stored in 0.9% normal saline at room temperature and used within two months of extraction to minimize changes in dentin properties. They were washed under running water and decoronated perpendicular to the long axis using a diamond disc (DZ, Darmstadt, Germany) under water cooling to obtain standardized 16 mm root segments, ensuring straight-line access and a flat reference surface. Roots were inspected under transmitted light and an operating microscope (×10-16 magnification) to exclude any craze lines or defects, with replacements sourced as needed.

Apical patency was established using size 10 and 15 K-files (Mani Inc., Tochigi, Japan) until the tip was just visible at the foramen, with the individual working length set 1 mm short by measuring with a Mini Endo Bloc (Dentsply Maillefer, Ballaigues, Switzerland). Teeth exhibiting apical sizes larger than #15, sclerosed dentin, or altered foramina were discarded. Teeth were randomly assigned to three equal groups (n = 20 each) based on instrumentation kinematics, using a computer-generated sequence with allocations concealed in sequentially numbered opaque envelopes until preparation commenced: Group I (hand), Group II (rotary ProTaper Next; Dentsply Maillefer, USA), and Group III (reciprocating WaveOne Gold; Dentsply Sirona, USA). All preparations were performed by a single endodontist with more than three years of experience, who was calibrated on the instrumentation protocols using five pilot specimens per group prior to the main experiment.

In Group I (hand instrumentation), canals were shaped using stainless steel K-files (#15-#35) in a reaming motion to working length, followed by the balanced-force technique with a #40 file as the master apical file, then step-back (#45-#55 at 1-mm increments). Recapitulation with a #15 file was done between files. During canal preparation, each canal received a total of 10 mL of 3% sodium hypochlorite, delivered in 1 mL aliquots between instruments at an approximate flow rate of 0.2 mL/s using a side‑vented 30‑gauge needle. The needle was positioned 1 mm short of the working length without binding and was moved with a gentle up‑and‑down motion to minimize the risk of extrusion. A final rinse protocol consisting of 1 mL of 17% ethylenediaminetetraacetic acid (EDTA) followed by 3 mL of distilled water was applied in all groups, using the same needle type, insertion depth, and delivery rate as described above, and canals were dried with paper points [12].

In Group II (rotary), the glide path was confirmed with a #15 K-file before sequencing ProTaper Next M-Wire files (X1: 17/.04, X2: 25/.06, X3: 30/.07, X4: 40/.06; all to working lengths) via the X-Smart Plus motor (Dentsply Maillefer, Ballaigues, Switzerland) per manufacturer’s settings (torque/speed: X1-X2 300 rpm/2 Ncm; X3-X4 250-300 rpm/4 Ncm). Irrigation and drying protocols followed the same as in hand Group I.

In Group III (reciprocating), canals were shaped with WaveOne Gold Gold‑Wire files - primary (25/.07), medium (35/.06), and large (45/.05) - using the X-Smart Plus motor in reciprocation mode per manufacturer’s guidelines (150-300 rpm/adaptive torque). The irrigation protocol matched that of the previous groups.

After instrumentation, each root was carefully prepared for microscopic evaluation. Longitudinal grooves about 2 mm deep were made on the buccal and lingual surfaces using a diamond disc. Care was taken not to penetrate into the root canal space during grooving, so that artificial debris would not be introduced.

**Figure 1:**
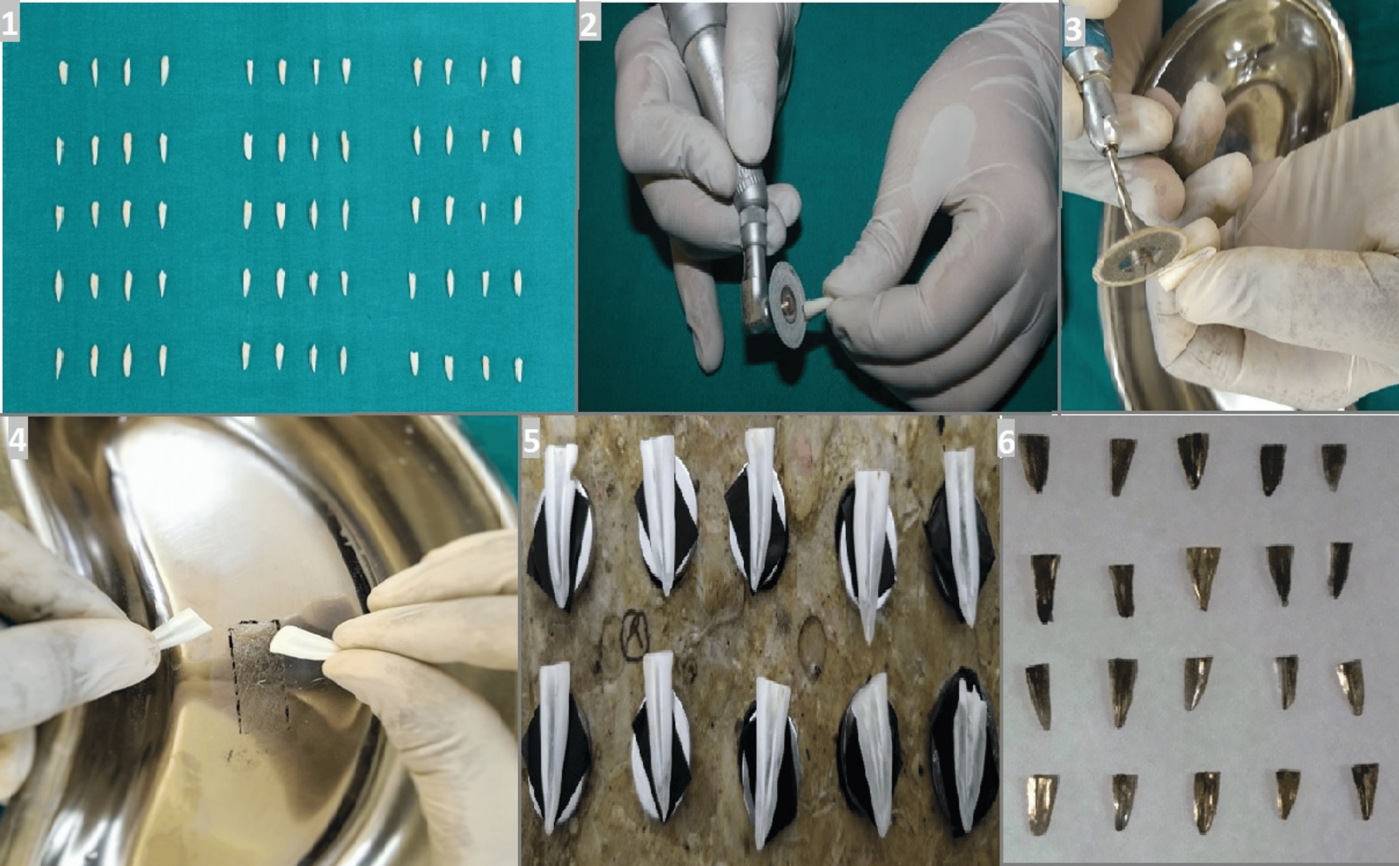
(1) Sixty tooth samples; (2) decoronation of the tooth samples; (3) creation of longitudinal grooves to split the samples into two halves; (4) samples after splitting; (5) mounted samples on coded metallic stubs; and (6) gold-sputtered samples.

Next, the roots were split lengthwise using a combination of a chisel and mallet (API, India) along with a straight handpiece (NSK Ltd., Tochigi, Japan) to obtain two halves. After longitudinal splitting, both halves of each root were inspected at low magnification, and the half providing an unobstructed view of the apical third (without cracks or detachment of dentin) was selected for analysis according to predefined criteria. If both halves fulfilled these criteria, the buccal half was chosen by convention to avoid selection bias. Each selected specimen was appropriately coded, dehydrated to remove any residual moisture, and then mounted securely on metallic stubs. The samples were then sputter-coated with a thin gold layer to make them electrically conductive for SEM examination (Figure 1).

The specimens were examined under an SEM (EVO 40 XVP, Carl Zeiss, Oberkochen, Germany) operated in high-vacuum mode at 10 kV. Images of the apical third of the root canal wall were captured at ×1500 magnification using the microscope’s digital imaging system. Representative SEM images of the apical third were analyzed quantitatively using ImageJ version 1.53 (National Institutes of Health, Bethesda, MD, USA). Images were converted to 8‑bit grayscale, calibrated using the embedded scale bar (1 µm/pixel resolution), and a standardized 200 × 300 µm rectangular region of interest was drawn centered on the canal lumen. Smear layer coverage was quantified by applying the Otsu automatic thresholding method to segment debris-covered dentin from clean surfaces within the region of interest, with results expressed as percentage area affected. Identical threshold settings were maintained across all images.

To ensure unbiased assessment, all SEM images were scored by a blinded examiner using standardized and validated criteria described by Prati et al. (2004) [13]. Two independent evaluators, blinded to treatment groups, scored five representative images for calibration prior to the main analysis, resolving discrepancies through consensus on borderline cases. Inter‑examiner agreement for apical smear‑layer, pulpal debris, and inorganic debris scores was assessed using Cohen's weighted kappa coefficient (κ = 0.81, 95% CI: 0.72-0.90), indicating substantial reliability. The scoring system evaluated the smear layer, pulpal debris, and inorganic debris based on the amount of debris covering the dentinal surface as follows:

Smear layer assessment

**Figure 2:**
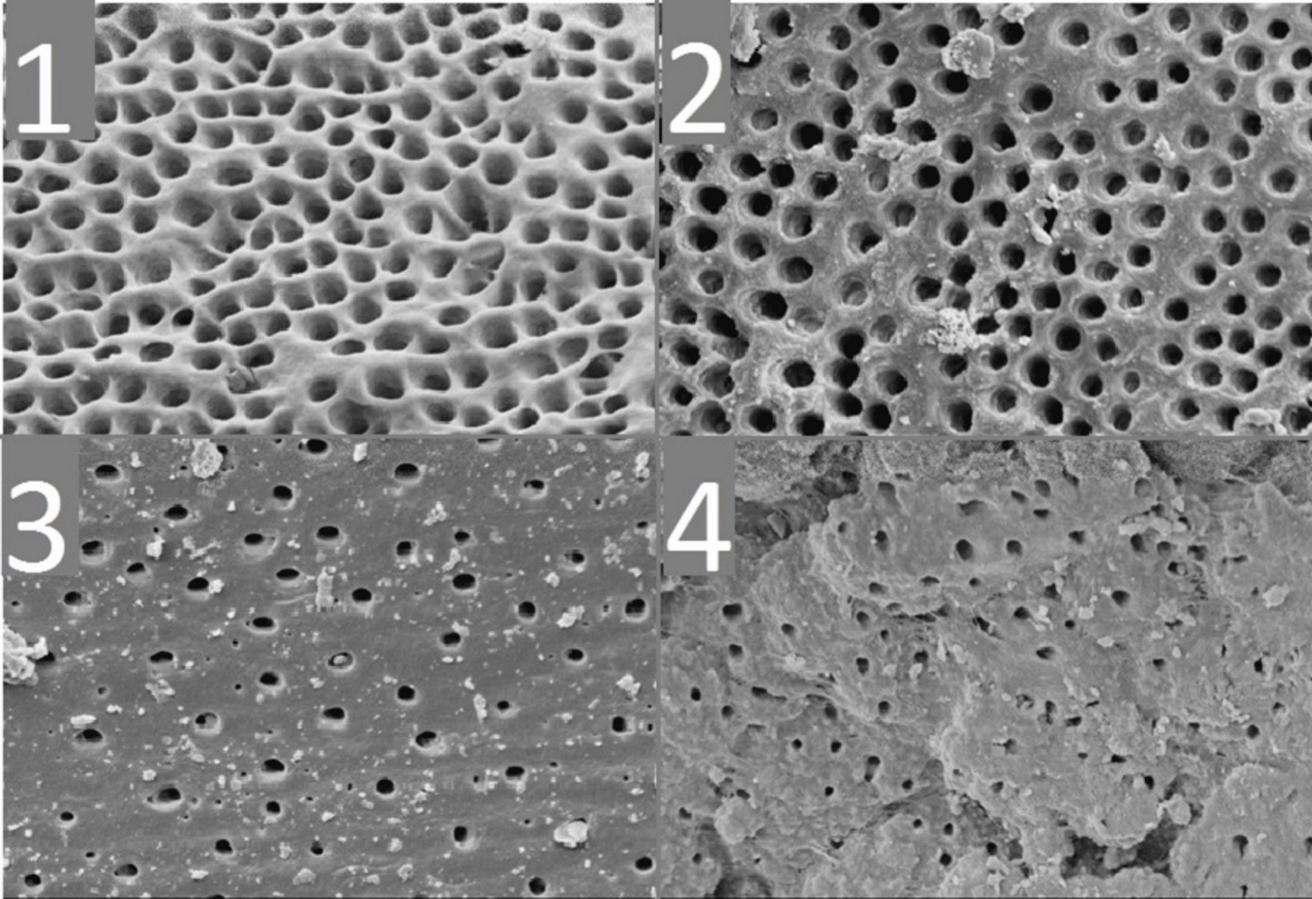
Smear layer scores: (1) Score 1; (2) Score 2; (3) Score 3; and (4) Score 4.

Score 1: No smear visible; over 75% of dentinal tubules fully open and clear; Score 2: Limited coverage; under 75% of tubules remain accessible; Score 3: Moderate presence; few tubules partially visible amid debris; Score 4: Thick, uniform layer sealing entire dentin surface (Figure 2).

Pulpal debris assessment

**Figure 3:**
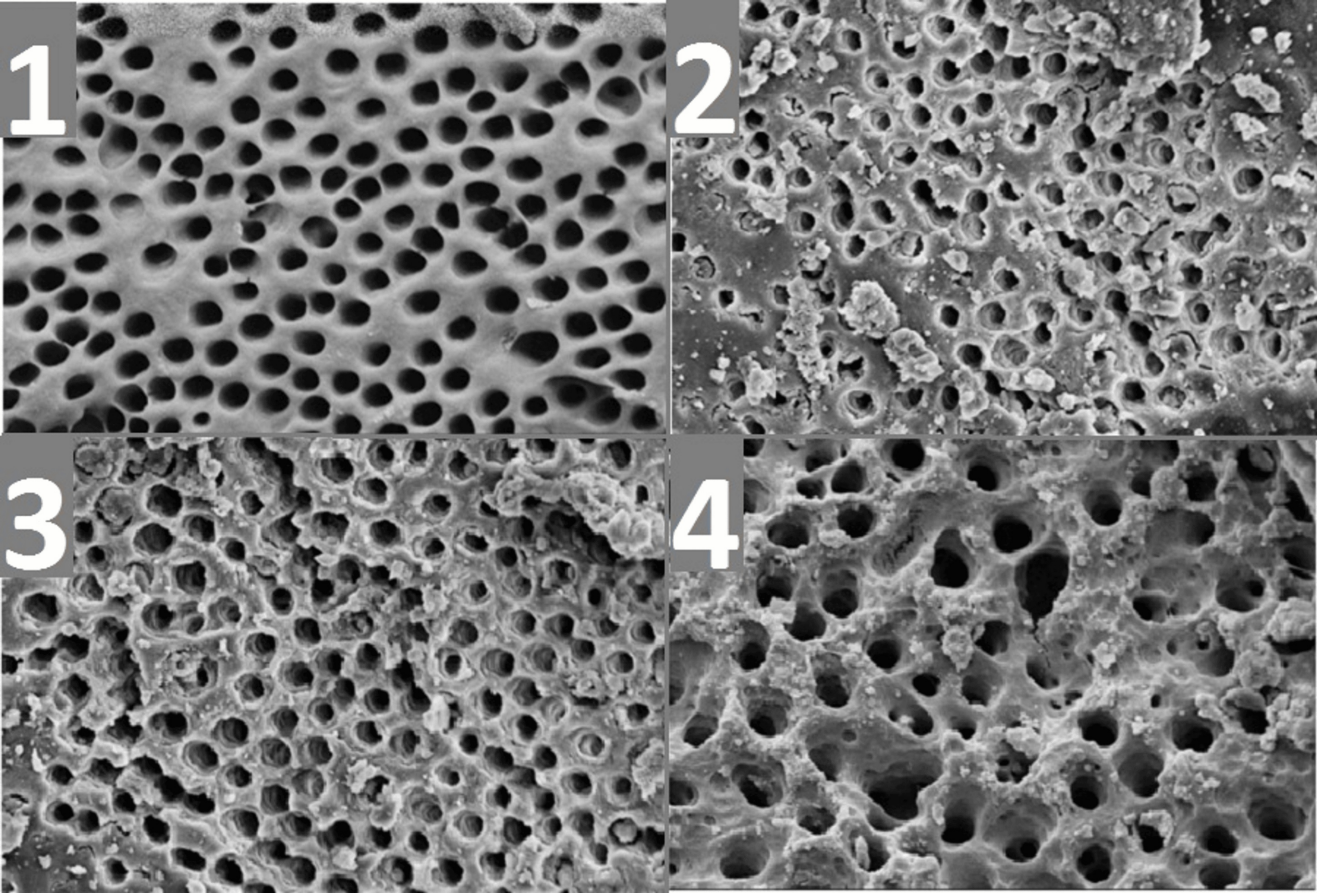
Pulpal debris scores: (1) Score 1; (2) Score 2; (3) Score 3; and (4) Score 4.

Score 1: None detected; Score 2: Trace fibrous remnants; Score 3: Noticeable scattered debris; Score 4: Dense collagen matrix throughout (Figure 3).

Inorganic debris assessment

**Figure 4:**
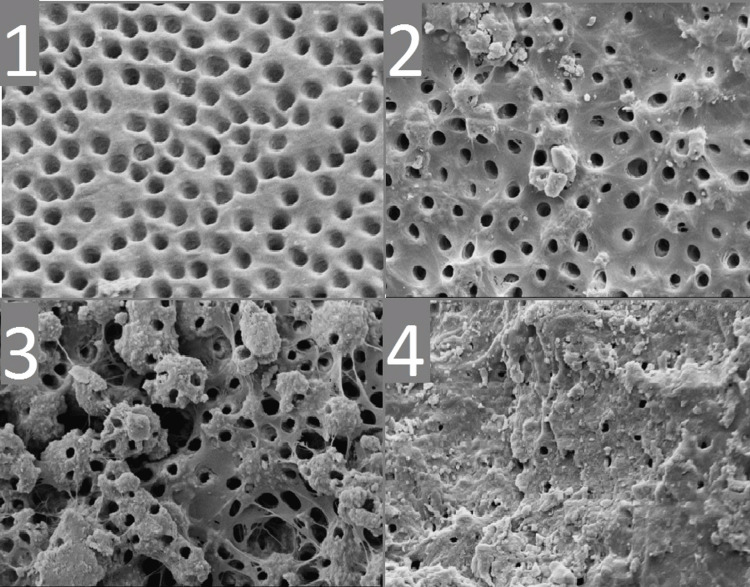
Inorganic debris scores: (1) Score 1; (2) Score 2; (3) Score 3; and (4) Score 4.

Score 1: Completely absent; Score 2: Scant traces; Score 3: Common scattered particles; Score 4: Heavy coverage across dentin (Figure 4).

Statistical analysis

Ordinal scores for smear layer, pulpal debris, and inorganic debris in the apical third were analyzed using non-parametric methods due to non-normal distribution (Shapiro-Wilk test, p < 0.05 for all groups and outcomes) and unequal variances (Levene's test, p < 0.05). The Kruskal-Wallis H test was employed to compare medians across the three independent groups (hand, rotary, reciprocating), with the test statistic H calculated as $H = \frac{12}{N(N+1)} \sum \left( \frac{R_i^2}{n_i} \right) - 3(N+1)$, where N = 60 total samples, R_i_ = sum of ranks for group i, and n_i_ = 20 per group. This was followed by post-hoc pairwise Mann-Whitney U tests (Wilcoxon rank-sum), with $U = n_1 n_2 + \frac{n_1(n_1 + 1)}{2} - R_1$, standardized as $Z = \frac{U - \mu_U}{\sigma_U}, \quad \mu_U = \frac{n_1 n_2}{2}, \quad \sigma_U = \sqrt{\frac{n_1 n_2 (N+1)}{12}}$, applying Bonferroni correction (α = 0.05/3 = 0.017) for multiple comparisons to control family-wise error rate. Effect sizes were reported as $r = \frac{Z}{\sqrt{N}}$. Data were analyzed using IBM SPSS Statistics for Windows, Version 27 (Released 2019; IBM Corp., Armonk, New York, United States).

## Results

Smear layer scores

Descriptive statistics revealed distinct differences in smear layer scores across groups (Table 1). The hand instrumentation group exhibited the lowest mean score (3.00 ± 0.92), while rotary (4.60 ± 0.50) and reciprocating (4.40 ± 0.50) groups showed substantially higher values. The Kruskal-Wallis test confirmed overall significant differences (H = 33.84, p < 0.001, df=2, χ^2^ approximation valid as n_i ≥5 and no ties >20%).

**Table 1:** Comparison of smear layer, pulpal debris, and inorganic debris scores among instrumentation groups *p < 0.001 (all main effects statistically significant)

Parameter	Group	Mean ± SD	Mean Rank	Kruskal-Wallis (H)	p-value	Post-hoc Comparison (Mann-Whitney U)
Smear layer scores	Hand (n = 20)	3.00 ± 0.92	12.10	33.84	<0.001*	Hand vs. Rotary (Z = -4.79, p < 0.001); Hand vs. Reciprocating (Z = -4.44, p < 0.001); Rotary vs. Reciprocating (Z = -1.25, p = 0.289)
	Rotary (n = 20)	4.60 ± 0.50	28.90			
	Reciprocating (n = 20)	4.40 ± 0.50	25.00			
Pulpal debris scores	Hand (n = 20)	2.40 ± 0.50	12.10	29.45	<0.001*	Hand vs. Rotary (Z = -4.78, p < 0.001); Hand vs. Reciprocating (Z = -4.82, p < 0.001); Rotary vs. Reciprocating (Z = -0.71, p = 0.529)
	Rotary (n = 20)	3.85 ± 0.81	28.90			
	Reciprocating (n = 20)	3.65 ± 0.59	27.00			
Inorganic debris scores	Hand (n = 20)	2.60 ± 0.50	10.50	96.06	<0.001*	Hand vs. Rotary (Z = -5.61, p < 0.001); Hand vs. Reciprocating (Z = -5.61, p < 0.001); Rotary vs. Reciprocating (Z = -1.25, p = 0.289)
	Rotary (n = 20)	4.60 ± 0.50	30.50			
	Reciprocating (n = 20)	4.40 ± 0.50	29.00			

Post-hoc Mann-Whitney U tests identified superior performance of hand instrumentation over rotary (Z = -4.79, p < 0.001) and reciprocating (Z = -4.44, p < 0.001) groups, with no difference between rotary and reciprocating systems (Z = -1.25, p = 0.289).

Pulpal debris scores

Pulpal debris scores followed a similar pattern (Table 1). Hand instrumentation yielded the lowest mean (2.40 ± 0.50), compared to rotary (3.85 ± 0.81) and reciprocating (3.65 ± 0.59) groups. Kruskal-Wallis analysis demonstrated significant intergroup variation (H = 29.45, p < 0.001, df = 2). Pairwise comparisons confirmed hand superiority versus rotary (Z = -4.78, p < 0.001) and reciprocating (Z = -4.82, p < 0.001) groups, while rotary and reciprocating showed comparable results (Z = -0.71, p = 0.529).

Inorganic debris scores

Inorganic debris accumulation was most pronounced in mechanized groups (Table 1). Hand files achieved the lowest score (2.60 ± 0.50), significantly below rotary (4.60 ± 0.50) and reciprocating (4.40 ± 0.50) means. The Kruskal-Wallis test indicated robust differences (H = 96.06, p < 0.001, df = 2). Mann-Whitney U tests corroborated hand instrumentation's advantage over rotary (Z = -5.61, p < 0.001) and reciprocating (Z = -5.61, p < 0.001) techniques, with insignificant differences between rotary and reciprocating methods (Z = -1.25, p = 0.289) (Table 1).

Across all debris parameters, hand instrumentation consistently demonstrated superior canal wall cleanliness in the apical third compared to rotary and reciprocating NiTi systems, which performed equivalently.

## Discussion

Hand instrumentation's superiority arises from stainless steel K-files' rigidity, promoting aggressive lateral dentin scraping and coronal debris extrusion via reaming and balanced-force motions, unlike superelastic NiTi files prone to apical burnishing. Reddy et al. [1] attributed comparable manual NiTi advantages over ProTaper rotary to hand files' disruption of smear plugs within dentinal tubules. Manjunatha et al. [14] similarly documented manual superiority over rotary techniques with EDTA irrigation via SEM, aligning with current findings of enhanced apical cleaning through mechanical agitation.

These results are consistent with histological evaluations showing stainless steel K-files leaving minimal debris in the apical third compared to ProTaper rotary files, attributed to fewer rotations and less apical compaction in manual techniques [3]. Similarly, studies comparing hand K-files to NiTi systems report better debridement scores (p < 0.05) due to tactile control enabling targeted filing in curved apices [15].

Rotary ProTaper Next and reciprocating WaveOne Gold performed equivalently due to shared limitations in apical ramifications, where continuous rotation compacts dentin while reciprocation reduces fatigue but fails to disrupt vapor lock impeding irrigant renewal. Poggio et al. noted Mtwo rotary leaving less smear than reciprocating overall, yet both trailed hand techniques, mirroring these kinematic constraints [5]. Adsare et al. observed WaveOne excelling coronally but equaling rotary apically, confirming zone-specific mechanized deficiencies [2]. In oval canals, full-sequence ProTaper and reciprocating single-file showed similar apical debris but less than primary WaveOne, linked to file design and motion failing to access fins/isthmuses; however, both lagged manual scraping [16]. These results corroborate Robinson et al.'s [17] micro-CT findings of rotary extruding more debris than reciprocation, yet both are inferior to manual methods amid persistent apical uncleanliness.

Stainless steel files generate more cutting strokes per insertion through circumferential filing, effectively loosening packed debris from lateral walls and fins, whereas rotary motion in ProTaper Next often packs chips apically due to continuous helical advancement [18]. The rigidity of stainless steel enables deeper tubule penetration and debris dislodgement, unlike NiTi's elasticity, causing smear burnishing, especially with 3% NaOCl/EDTA without activation [19].

Clinical implications of this study include the observation that while hand instrumentation demonstrated superior apical cleanliness in this in vitro model, rotary and reciprocating NiTi systems provide time savings (up to five minutes per canal) and reduce operator fatigue, with comparable long-term success rates in primary teeth (risk ratio (RR) = 1.01; moderate evidence) [20]. Clinicians may prioritize hand files for narrow or calcified apices to achieve optimal debridement, while hybridizing with NiTi systems for efficiency in straighter canals. Enhanced irrigation protocols could address NiTi limitations, though tactile feedback remains crucial for treatment prognosis. Future clinical trials linking SEM debris scores to healing outcomes are needed [21].

Nevertheless, some limitations should be considered when interpreting the findings. The use of a single experienced operator ensured consistency of the instrumentation procedures but may also introduce operator-related bias and limit extrapolation to clinicians with different levels of training or technique. Furthermore, only single-rooted premolars with relatively straight canals were included, which restricts generalizability to multi-rooted teeth or canals with greater curvature and more complex anatomy, where debris and smear-layer removal may be more challenging. Smear-layer assessment relied on subjective SEM scoring, and inter-rater reliability statistics were not calculated, so the degree of agreement between examiners cannot be quantified. Although the use of validated scoring criteria and examiner blinding reduces some subjectivity, the absence of explicit reliability metrics may influence the precision of the reported differences between groups.

Another limitation is that the analysis was restricted to two-dimensional SEM images of selected root halves, without three-dimensional volumetric confirmation. Consequently, the true spatial distribution of remaining debris and smear layer within the entire canal system, particularly within isthmuses, lateral canals, and irregularities, could not be fully characterized.

Future studies incorporating micro-computed tomography (micro-CT) could quantitatively assess volume and distribution of residual debris and dentin removal in three dimensions, thereby reducing sampling bias inherent to single-plane SEM imaging. Combining micro-CT with standardized SEM scoring and formal inter-examiner reliability analysis would provide a more comprehensive picture of cleaning efficacy and improve the reproducibility of the findings. In addition, clinical or ex vivo studies that compare outcomes in teeth with more complex anatomies and multiple operators could clarify how these instrumentation systems perform under realistic clinical variability. Such work would help determine whether the differences observed in this controlled in vitro setting translate into improved clinical debridement and long-term treatment success.

## Conclusions

Within the limits of this in vitro study, hand instrumentation using stainless steel K-files achieved significantly lower smear-layer, pulpal debris, and inorganic debris scores in the apical third compared to rotary ProTaper Next and reciprocating WaveOne Gold NiTi systems (p < 0.001, Kruskal-Wallis test, Post-hoc Mann-Whitney U tests). These findings suggest greater cleaning effectiveness of hand files in this standardized model with 3% NaOCl, 17% EDTA irrigation, despite metallurgical improvements in NiTi rotary systems. However, the controlled experimental conditions cannot fully replicate clinical variations in irrigation dynamics, canal anatomy, or operator technique.

Clinical healing outcomes were not assessed in this study. Future research using micro-CT for three-dimensional debris quantification and prospective clinical trials will be needed to determine if these in vitro cleaning differences correlate with improved long-term endodontic success.
